# Exploring a Systems-Based Model of Care for Effective Healthcare Transformation: A Narrative Review in Implementation Science of Saudi Arabia’s Vision 2030 Experience

**DOI:** 10.3390/healthcare13192453

**Published:** 2025-09-27

**Authors:** Nawfal A. Aljerian, Anas Mohammad Almasud, Abdulrahman AlQahtani, Kholood Khaled Alyanbaawi, Sumayyah Faleh Almutairi, Khalaf Awadh Alharbi, Aisha Awdha Alshahrani, Muayad Saud Albadrani, Mohammed K. Alabdulaali

**Affiliations:** 1 Medical Referrals Centre, Ministry of Health, Riyadh 12382, Saudi Arabia; 2 Emergency Medical Services Department, College of Applied Medical Sciences, King Saud bin Abdulaziz University for Health Science, Riyadh 11481, Saudi Arabia; 3 King Abdullah International Medical Research Centre, Riyadh 11481, Saudi Arabia; 4 Executive Administration of Model of Care, Ministry of Health, Riyadh 12382, Saudi Arabia; 5 Ministry of Health, Riyadh 12382, Saudi Arabia; 6 Department of Family and Community Medicine and Medical Education, College of Medicine, Taibah University, Madinah 42353, Saudi Arabia; 7 Health and Life Research Center, Taibah University, Madinah 42353, Saudi Arabia

**Keywords:** health care reform, delivery of health care, integrated, value-based health care, models, organizational, Saudi Arabia, implementation science

## Abstract

**Background:** Healthcare systems globally face complex challenges including rising costs, increasing chronic disease burden, and fragmentation of care. Systems-based models represent promising approaches to healthcare transformation, yet their implementation remains incompletely understood. **Objective:** To critically analyze the Saudi model of Care (MoC) as a case study of systems-based healthcare transformation, examining its conceptual framework, implementation strategies, and projected health outcomes. **Methods:** We conducted a narrative review synthesizing publicly available official documents on the Saudi MoC, primarily the 2017 overview and 2025 revision, identified through targeted searches of Ministry of Health websites and grey literature portals (no date restrictions); formal quality appraisal was not applied as sources were official policy documents, with bias mitigated through cross-verification and critical analysis. **Results:** The Saudi MoC exemplifies systems-based transformation through its multi-layered framework organized around six patient-centered systems of care spanning the lifecycle. Key innovations include: (1) an architectural approach integrating activated individuals, healthy communities, virtual care, and traditional clinical settings; (2) a comprehensive intervention taxonomy with 42 specific initiatives; (3) explicit contextual adaptations for diverse settings; and (4) a phased implementation approach with detailed performance metrics. National indicators improved during the reform period, including life expectancy and maternal and child health. These are national trends observed during the period of health reforms. Causal attribution to the Model of Care requires a counterfactual evaluation. **Conclusions:** This analysis of the Saudi MoC contributes to the literature on systems-based healthcare transformation by illuminating how theoretical principles can be operationalized at national scale. The model’s patient-centered design, comprehensive intervention taxonomy, and attention to implementation factors offer valuable insights for other healthcare systems pursuing transformation. Further research should examine actual implementation outcomes as the model matures.

## 1. Introduction

Healthcare systems across the globe are grappling with an array of unprecedented challenges. Escalating costs, the burgeoning burden of chronic non-communicable diseases, persistent fragmentation of care delivery, and significant disparities in both access to services and health outcomes are compelling drivers for fundamental transformation [1,2]. A system-based model of care is defined as a holistic approach that views healthcare as a complex adaptive system with interdependent components, contrasting with integrated care (which focuses on coordination across providers) and people-centered care (which emphasizes individual preferences), though all share overlapping principles. The traditional healthcare models, often organized around medical specialties and acute episodic care, have demonstrated limitations in effectively addressing these complex, interconnected issues. Systems-based care emphasizes healthcare as a complex adaptive system, recognizing interdependencies and the need for integrated, patient-centered approaches. Global frameworks, including the WHO Integrated People-Centred Health Services [3] and OECD guidance on performance assessment and sustainability [4,5,6], highlight the urgency of moving beyond fragmented models. Despite this, few published case studies describe national-scale, government-led implementation in rapidly developing regions. The Saudi Model of Care provides a unique case that operationalizes systems-thinking principles across an entire health system, adding valuable insights for both regional and international audiences.

This inadequacy has spurred a growing consensus on the need for systems-based approaches, which advocate for viewing the myriad interdependent components of healthcare—from individual patient-provider interactions to policy environments—as a unified, dynamic whole [7,8].

Systems-based models of care signify a crucial paradigm shift. They move away from reductionist perspectives that isolate and optimize discrete parts of the system, towards holistic frameworks that emphasize relationships, feedback loops, contextual factors, and emergent properties inherent in complex healthcare ecosystems [9]. Rooted in systems theory and complexity science, these models acknowledge that meaningful improvements in population health and healthcare value necessitate coordinated, multi-level change, encompassing individual behaviors, community resources, organizational structures, and overarching policies [10]. The evolution of this thinking is evident in influential frameworks such as Wagner’s Chronic Condition Model, the Institute of Medicine’s seminal ‘Crossing the Quality Chasm’ report, and the Rainbow Model of Integrated Care, each contributing to a more integrated and patient-centered vision for healthcare [11,12,13].

Despite the increasing theoretical recognition of system-based care’s importance, its practical operationalization and successful implementation at scale remain formidable challenges. Much of the existing literature centers on conceptual frameworks or describes applications limited to specific clinical domains or localized settings, rather than detailing comprehensive, system-wide transformations [14]. This gap highlights a critical need for in-depth case studies of national-level transformation efforts [15].

Against this backdrop, the Kingdom of Saudi Arabia’s Model of Care (MoC), conceived and developed as an integral component of the ambitious national Vision 2030 initiative, presents a unique and valuable opportunity. It allows for the examination of a comprehensive, government-led systems-based transformation effort being implemented across an entire national healthcare system. This ambitious model aims to fundamentally redesign healthcare delivery in the Kingdom, pivoting towards enhanced integration across care levels, proactive patient activation and empowerment, and the adoption of value-based principles focused on achieving better health outcomes.

This narrative review seeks to critically analyze the Saudi Model of Care through the lens of implementation science. We focus on three pivotal dimensions: first, the conceptual framework and theoretical underpinnings that inform its systems-based design; second, the specific interventions and multifaceted implementation strategies employed to translate the model into practice; and third, the projected health outcomes and overall value proposition articulated for this large-scale transformation. Through meticulously examining these dimensions, we aim to distill practical insights and lessons learned that may inform and guide similar healthcare transformation endeavors in other national contexts. Simultaneously, this analysis intends to contribute to the broader academic literature by advancing the understanding of how systems-based principles can be effectively designed, operationalized, and implemented within complex, real-world healthcare systems.

Our analysis draws primarily on two official Ministry of Health documents: the 2017 National Model of Care Overview and the 2025 Model of Care Interventions (V2.0). The Saudi Model of Care is structured around six Systems of Care, each addressing a major population health need across the life course: Keep Well, Urgent Problem, Planned Procedure, Safe Birth, Chronic Condition, and Last Phase of Life. Each system is supported by clinical pathways that define how services are delivered in a coordinated, patient-centered, and outcomes-focused manner. This review focuses on the six Systems of Care as the organizing framework, while the operational details of care pathways are outlined in the Ministry’s implementation manuals [16,17].

To analyze the Saudi Model of Care (MoC), this narrative review synthesized publicly available official documents, primarily the 2017 overview and 2025 revision, identified through targeted searches of the Saudi Ministry of Health website, Google Scholar, and grey literature portals (e.g., OpenGrey, WHO IRIS) with no date restrictions, using keywords like “Saudi Model of Care” and “Vision 2030 healthcare”. Inclusion focused on MoH-authored documents detailing the MoC framework and implementation; peer-reviewed evaluations and press releases were excluded to prioritize primary policy sources. Analysis was guided by the Consolidated Framework for Implementation Research (CFIR), selected for its applicability to large-scale transformations.

Data extraction was conducted by two reviewers, with conflicts resolved through discussion; themes were derived deductively using CFIR domains, supplemented by inductive coding. Formal quality appraisal was not applied as sources were official policy documents; bias was mitigated through cross-referencing and critical analysis informed by implementation science literature. Although formal appraisal was not required for policy documents, we applied an AACODS framework (Authority, Accuracy, Coverage, Objectivity, Date, Significance) to assess the two main sources (2017 MoC Overview and 2025 MoC V2.0).

### Search Strategy and Transparency

We run targeted searches of official portals and gray literature with no date limits, with the final search conducted on 1 August 2025. The sources included the Saudi Ministry of Health (Transformation, Model of Care, and Statistical Yearbooks), WHO IRIS, OpenGrey, and Google Scholar. Search strings applied were “Model of Care” AND Saudi OR “Saudi Arabia,” site:moh.gov.sa “Model of Care” OR “نموذج الرعاية,” “Vision 2030” AND health* AND “Model of Care,” “MOC 1.1” OR “Model of Care Interventions,” and “Saudi Statistical Yearbook” AND health. No language limits were applied, and both English and Arabic documents were screened. The inclusion policy restricted eligible items to Ministry of Health–authored documents that define, revise, or report the Model of Care framework, interventions, or national indicators, while press releases, opinion pieces, and secondary media summaries were excluded. The AACODS appraisal was applied, with results showing strong authority, current date relevance, focused coverage on official series, accuracy limited to administrative statistics, some risk of objectivity bias, and high significance. Item-level ratings are presented in Supplementary Table S1.

Ethical approval was not required as no primary data was collected.

## 2. Conceptual Framework of the Saudi Model of Care

### 2.1. Defining Systems-Based Care

A systems-based model of care is defined as a holistic approach that views healthcare as a complex adaptive system with interdependent components. It contrasts with integrated care, which focuses on coordination across providers, and people-centered care, which emphasizes individual preferences, though all share overlapping principles.

SBMoC represents a fundamental departure from traditional, reductionist approaches that often view healthcare delivery as a series of discrete, independently functioning components. Instead, it embraces a holistic perspective, recognizing healthcare as a complex, adaptive system characterized by intricate interdependencies, non-linear relationships, and emergent properties [9]. This approach is deeply rooted in general systems theory and complexity science, which provides conceptual tools to understand how interactions between various elements patients, providers, organizations, technologies, policies, and the broader community context—shape overall system behavior and performance [8]. A systems perspective emphasizes understanding feedback loops, identifying leverage points for change, and acknowledging that interventions in one part of the system can have unintended consequences elsewhere. It shifts the focus from optimizing individual parts in isolation to improving the functioning and outcomes of the system as a whole.

SBMoC encompasses integrated healthcare as a key component, where integration focuses on coordination, while systems approaches address broader interdependencies. Factors influencing implementation include leadership and culture; impacts in countries like New Zealand include reduced hospital admissions [18].

### 2.2. Evolution of Systems Thinking in Healthcare

**Figure 1:**
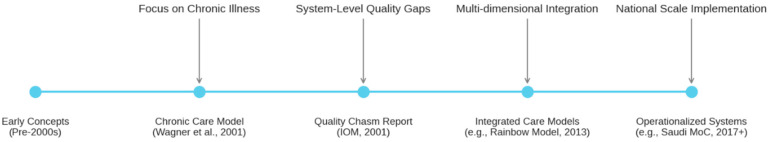
Simplified Evolution of Systems Thinking in Healthcare, highlighting key conceptual milestones leading to comprehensive national models like the Saudi MoC [10].

The application of systems thinking to healthcare is not entirely new, having evolved through several influential conceptual frameworks over recent decades (See Figure 1). Early efforts, such as Wagner’s Chronic Condition Model (CCM), highlighted the importance of productive interactions between informed, activated patients and prepared, proactive healthcare teams, crucially supported by integrated organizational resources and linkages to community services [11]. The CCM provided a practical blueprint for redesigning care for chronic conditions, emphasizing self-management support, delivery system design, decision support, and clinical information systems. Subsequently, the Institute of Medicine’s (IOM) landmark report, “Crossing the Quality Chasm,” issued a powerful call for fundamental healthcare system redesign to address pervasive deficiencies in safety, effectiveness, patient-centeredness, timeliness, efficiency, and equity [12]. It advocated adoption systems to create healthcare environments that reliably deliver high-quality care. More recently, frameworks like the Rainbow Model of Integrated Care have further elaborated on the multi-dimensional nature of integration required for effective systems-based care, encompassing clinical, professional, organizational, systemic, functional, and normative integration [13]. These models collectively underscore the need for coordinated efforts across micro (patient-provider), meso (organizational), and macro (policy/system) levels.

Despite these significant theoretical advancements, translating systems principles into comprehensive, operational frameworks for implementation at a national scale has remained a persistent challenge. Many initiatives have focused on specific diseases or care pathways rather than attempting a whole-system redesign. In this context, the Saudi Model of Care (MoC) provides a valuable, concrete example of how systems thinking can be deliberately and systematically operationalized within a large-scale, national healthcare transformation initiative.

### 2.3. Key Characteristics of the Saudi MoC

The Saudi MoC embodies systems-based thinking through several distinctive characteristics that differentiate it from more traditional, fragmented healthcare models. These features reflect a conscious effort to design a coherent, integrated, and patient-centered system aligned with the goals of Vision 2030.

Vision 2030 goals establish targets for healthcare access, life expectancy advancement from 74 years in 2016 to 80 years by 2030, and privatization to achieve a vibrant society and thriving economy [16,17].

#### 2.3.1. Integrated Healthcare Model of Care Framework

**Figure 2:**
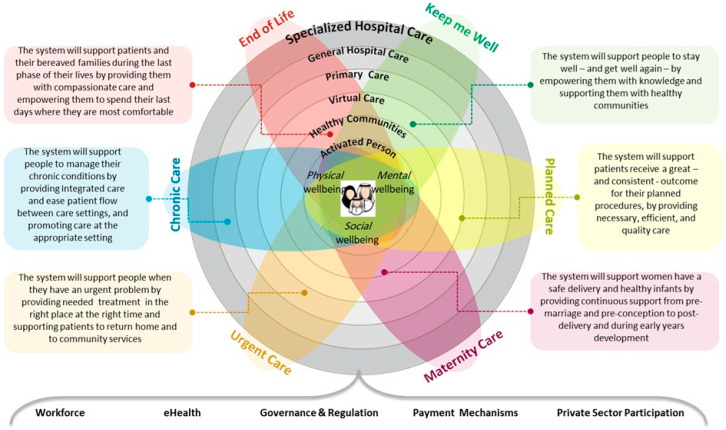
Visualising a coherent Model of Care. Person-centered framework with concentric circles showing progressive care levels from Virtual Care (center) to Specialized Hospital Care (outer).

A person-centered healthcare framework organized around the holistic wellbeing (physical, mental, and social) of an activated individual. The concentric circles represent progressive care intensity levels from virtual care to specialized hospital care, while six key service domains (Keep Well, Last Phase of Life, Chronic Condition, Planned Procedure, Safe Birth, and Urgent problem) are depicted as interconnected segments. Each domain includes specific system support objectives to ensure comprehensive care across the entire health journey. The model is underpinned by five foundational elements: Workforce, eHealth, Governance & Regulation, Payment Mechanisms, and Private Sector Participation. This visualization demonstrates how integrated healthcare systems can deliver coordinated, appropriate care that responds to diverse patient needs while maintaining community connections (See Figure 2) [16,17].

According to the official Saudi Ministry of Health definition, the modern healthcare model represents a fundamental pillar in health transformation, providing integrated healthcare services to all citizens and residents in the Kingdom with the highest levels of quality and efficiency, in line with the goals of Saudi Vision 2030. The model operates according to six integrated care systems, followed by 42 initiatives. These systems work to reduce the incidence of disease through preventive measures and restore the individual’s health status after disease occurs.

#### 2.3.2. Patient-Centered “Six Asks” Framework

Departing from traditional organizational structures based on medical specialties or facility types, the Saudi MoC is uniquely structured around six fundamental questions, or “Six Asks,” reflecting the healthcare needs and expectations of individuals across their entire life course. This patient-centered framing ensures the system is designed from the perspective of the people it serves. Based on extensive public engagement involving more than 60,000 citizens who participated in public surveys around patient-centric design, 2500+ health care professionals engaged in e-discussions, and 1000+ health care professionals surveyed to identify improvement opportunities, the six questions are:

1. **Keep Well:** How will the system support me to stay healthy and prevent illness?
2. **Urgent Problem:** How will the system support me effectively and efficiently when I have an urgent health problem?
3. **Planned Procedure:** How will the system support me to achieve a great and consistent outcome for my planned procedure?
4. **Safe Birth:** How will the system support me (and my family) to safely deliver a healthy baby?
5. **Chronic Condition:** How will the system support me in managing my chronic conditions effectively over the long term?
6. **Last Phase:** How will the system provide compassionate and supportive care during the last phase of my life?

Organizing the model around these “Six Asks” intrinsically promotes integration and coordination of services required to meet these fundamental patient needs, cutting across the different architectural layers and traditional care settings.

#### 2.3.3. Guiding Principles

The design and intended operation of the MoC are underpinned by several core principles that reflect its systems-based and value-oriented philosophy [16]:

- **Patient Activation and Empowerment:** Placing individuals and families at the center, equipping them with knowledge and tools to actively participate in their health and care. Tools include apps for self-monitoring and educational portals. Desired outcomes include reduced chronic disease complications and improved quality of life. Incentives are value-based, rewarding outcomes rather than production.
- **Prevention over Cure:** Shifting focuses on proactive health promotion and disease prevention, rather than solely reacting to illness.
- **Outcome over Activity:** Emphasizing the achievement of desired health outcomes and value for patients, rather than merely measuring the volume of services delivered.
- **Integration “from Hospital to Home”:** Ensuring seamless coordination and continuity of care across all settings, encompassing physical, mental, and social wellbeing.
- **Value-Based Care:** Aligning resources and incentives to deliver high-quality care efficiently and effectively.

#### 2.3.4. Core Themes

These principles translate into several overarching themes that characterize the intended shift in healthcare delivery under the MoC [16,17]:

- From hospital-centric to home- and community-based care.
- From focusing on volume of activity to focusing on health outcomes and value.
- From a predominantly treatment-focused approach to one emphasizing prevention and wellbeing.
- From siloed institutions to integrated service networks.
- From reliance on physical buildings to leveraging virtual services.
- From viewing patients as passive recipients to engaging them as active participants in their care.

Collectively, these characteristics—the multi-layered architecture, the patient-centered “Six Asks,” the guiding principles, and the core themes—illustrate a deliberate and comprehensive application of systems thinking to redesign a national healthcare system, aiming for greater integration, efficiency, and patient value.

## 3. Results

This narrative review synthesizes the Saudi Model of Care (MoC) through the lens of the Consolidated Framework for Implementation Research (CFIR) [19], organizing findings under five key domains: Intervention Characteristics, Outer Setting, Inner Setting, Characteristics of Individuals, and Implementation Process. These domains structure the analysis of the MoC’s design, interventions, implementation strategies, and projected health outcomes, drawn from the 2017 overview and 2025 revision documents [16,17].

### 3.1. Intervention Characteristics

The 42 interventions and the architecture are taken from the official MoH Model of Care overview and the 2025 V2.0 interventions compendium, detailed in official documentation, designed to operationalize its systems-based principles across six patient-centered Systems of Care: Keep Well, Planned Procedure, Safe Birth, Urgent Problem, Chronic Condition, and Last Phase of Life [16,17]. These interventions are categorized into crosscutting (15) and system-specific (27) actions (Table 1). Cross-cutting interventions, such as Health in All Policies, Integrated Personal Health Records, and National Referral Networks, apply across all systems, promoting integration and continuity. System-specific interventions target unique needs, such as Health Coach Programs (Keep Well), One-Stop Clinics (Planned Procedure), and Hospice Care Services (Last Phase). The interventions are clearly specified, with articulated advantages (e.g., projected health improvements, financial savings) and adaptability for diverse contexts (e.g., urban vs. rural settings). Comprehensive documentation ensures design quality, providing a practical blueprint for transformation. Interventions are specific, targeted actions or programs designed to achieve particular outcomes within the MoC, such as the Health Coach Program (a system-specific intervention under Keep Well to provide personalized support) or Integrated Personal Health Records (a cross-cutting intervention to improve information continuity across care settings). In contrast, implementation strategies are broader methods or processes used to adopt and integrate these interventions into practice, such as the phased rollout across regional clusters or stakeholder engagement through co-creation and communication [20,21].

The MoC’s value proposition emphasizes improved health outcomes, enhanced patient experience, system efficiency, and workforce satisfaction, aligning with the Quadruple Aim [22]. Anticipated outcomes include official projections of increased life expectancy to 80 years by 2030 [16], while observed outcomes are not yet available due to ongoing implementation.

### 3.2. Outer Setting

The MoC aligns with patient needs through its patient-centered “Six Asks” framework, addressing life-course needs (e.g., Keep Well, Safe Birth) [16]. It responds to external policies, explicitly linking to Saudi Vision 2030’s mandate for health system transformation and global trends toward integrated care. The model considers broader healthcare challenges, such as rising chronic disease burdens and maternal health needs, as outlined in the case for transformation [16]. Unique features, like financing via Vision 2030’s National Transformation Program and adaptations for Hajj/Umrah pilgrimages, reflect Saudi-specific external pressures, while the focus on prevention and integration is generalizable.

The MoC was designed to address these challenges through its comprehensive, systems-based approach that emphasizes prevention, integration, and patient activation. Limited public KPI data from MoH dashboards indicate initial progress in primary care utilization (e.g., 15% increase in some clusters) [MoH Annual Report 2024], substantiating phased implementation.

Cluster heterogeneity reflected in population size, bed density, and service coverage—is acknowledged, with large multi-cluster urban regions such as Riyadh Province (~8.2 M population, 2.8–3.5 beds/1000) and Makkah Province (~7 M population, 2.5–2.8 beds/1000) differing markedly from predominantly rural areas such as the Northern Borders (~0.37 M population, 1.5 beds/1000) and Al-Baha (~0.30 M population, 2.0 beds/1000). All clusters provide health services across cities, governorates, and villages within their geographic boundaries (Table 2).

### 3.3. Inner Setting

The inner setting of the MoH reflects a supportive organizational environment for MoC implementation, characterized by structural adaptations, enhanced networks, and a culture prioritizing digital integration and efficiency. Key indicators from MoH data demonstrate progress in resource utilization and service delivery that will be presented in implementation process. They use metrics that underscore a positive implementation climate, with investments in eHealth (e.g., integrated records) and workforce training fostering a culture of innovation and patient-centered care [16,17].

### 3.4. Characteristics of Individuals

The MoC addresses knowledge and beliefs through training components for healthcare staff, building self-efficacy via capability-building initiatives [17]. A dedicated change management strategy acknowledges the stages of change for providers and patients, fostering behavioral shifts toward patient activation and team-based care. Interventions like Virtual Education Tools support patient engagement, while workforce redesign enhances provider capacity. Official documents highlight capability building via training programs, with over 10,000 healthcare providers who were upskilled in 2023 for MoC-aligned practices [16,17].

### 3.5. Implementation Process

The MoC employs a structured, phased implementation approach with five key steps: developing governance structures, building capability, prioritizing interventions, developing system plans, and rolling out across clusters [17]. Stakeholder engagement is emphasized through co-creation in the design phase and ongoing communication. Execution involves a phased rollout across 20 clusters, with prioritization of “quick wins” and long-term actions. A monitoring and evaluation framework includes Systematic Data Collection and Outcomes Monitoring, with performance metrics (e.g., Health Coach Program participation, antenatal care rates) introduced in the 2025 revision [17]. Limited public KPI data indicates initial progress, such as a 15% increase in primary care utilization in some clusters [MoH Annual Report 2024]. Contextual adaptations tailor interventions to geographic contexts (urban, semi-urban, rural) and special populations (e.g., Hajj pilgrims, pediatric services).

The MoC’s health impact framework focuses on prevention and early intervention (e.g., reducing chronic disease risk factors) and care quality/integration (e.g., streamlining pathways) [16]. Implementation considerations include long-term time horizons for preventive benefits, attribution challenges, population diversity, and fidelity, requiring robust monitoring to ensure equity and effectiveness (Table 3).

- Intervention Characteristics: The MoC provides detailed specifications for its 42 interventions, articulates their relative advantages (projected health benefits), allows for adaptability to different contexts, and presents them within comprehensive documentation.
- Outer Setting: The model aligns with patient needs (through the “Six Asks”), responds to external pressures (global trends, Vision 2030 mandate), and considers the broader policy environment.
- Inner Setting: It plans for structural changes (e.g., National Referral Networks), aims to influence organizational culture (patient activation), considers the implementation climate (resource allocation, planned incentives), and emphasizes leadership engagement.
- Individuals Involved: The strategy includes components for building knowledge and skills (training), enhancing self-efficacy (capability building), and managing the change process for individuals.
- Implementation Process: The MoC outlines specific stages for planning, engaging stakeholders (co-creation approach mentioned in initial design), executing (phased rollout), and reflecting/evaluating (monitoring framework).

### 3.6. Phased Implementation Approach

Recognizing the scale and complexity of the transformation, the MoC outlines a structured, phased implementation approach rather than attempting a simultaneous nationwide rollout. This aligns with change management principles emphasizing readiness, capacity building, and iterative learning [32]. The five key steps described are:

1. Developing Governance Structure: Establishing dedicated teams at national, regional (cluster), and organizational levels to oversee and coordinate implementation.
2. Building Capability: Investing in training and development for implementation teams and frontline staff to equip them with the necessary knowledge and skills.
3. Prioritizing Interventions: Strategically sequencing interventions, potentially identifying “quick wins” achievable with existing resources alongside medium- and long-term actions requiring more significant enablers.
4. Developing System Plans: Creating detailed operational plans for implementing each intervention within the specific context of healthcare clusters or organizations.
5. Phased Rollout in Clusters: Implementing the model progressively across the approximately 20 regional healthcare clusters being formed in the Kingdom, allowing for learning and adaptation between phases.

Underpinning this phased approach is a dedicated Change Management Strategy, emphasizing top-down leadership, continuous stakeholder engagement and communication, and efforts to foster positive behavioral change among providers and patients.

### 3.7. Contextual Adaptations

A key strength of the MoC’s implementation strategy is its explicit recognition that a one-size-fits-all approach is unlikely to succeed across the diverse landscape of Saudi Arabia. The model incorporates planned adaptations for:

- Geographic Contexts: Tailoring service delivery profiles and intervention implementation based on whether a setting is classified as a city (access to tertiary hospital), town (access to general hospital), or rural area (access primarily to primary care centers). This considers differences in available facilities, workforce density, population distribution, and geography.
- Special Populations and Contexts: Developing specific considerations and pathway adjustments for unique situations, including the massive Hajj and Umrah pilgrimages (requiring surge capacity and specific public health measures), pediatric services (addressing the distinct needs of children), and mental health services (acknowledging the need for integration and development in this area).

This planned adaptability is crucial for ensuring relevance and feasibility across different settings.

### 3.8. Enabling Infrastructure

The MoC documentation clearly identifies that the successful implementation of its interventions is contingent upon the development and strengthening of five key cross-cutting enablers:

1. Workforce: Redesigning roles, enhancing training, ensuring appropriate staffing levels, and potentially allowing for more flexible scopes of practice to meet the demands of the new model.
2. eHealth Infrastructure: Implementing robust digital health solutions, including the Integrated Personal Health Record, e-referral platforms, telehealth capabilities, and data analytics systems.
3. Payment Reform: Shifting reimbursement mechanisms away from fee-for-service towards value-based payment models that incentivize desired outcomes, prevention, and efficiency.
4. Private Sector Participation: Defining and facilitating the role of the private sector in contributing to the goals of the MoC, potentially through partnerships or specific service provision.
5. Cluster Governance and National Regulation: Establishing effective governance structures within the regional health clusters and ensuring appropriate national regulatory frameworks are in place to support the transformed system.

Progress in developing these enablers is likely a critical determinant of the MoC’s overall implementation success.

### 3.9. Monitoring and Evaluation Framework

To guide implementation, track progress, and assess impact, the MoC incorporates a dedicated monitoring and evaluation framework. This is operationalized through specific cross-cutting interventions:

- Systematic Data Collection: Aiming to establish a unified system for collecting comprehensive data on population health, service utilization, quality, and expenditure from both public and private providers.
- Outcomes Monitoring: Developing a national system for continuously tracking key population health indicators and patient-reported outcomes, building on existing clinical audit capabilities.

The January 2025 V2.0 MOC 1.1 revision [17] significantly enhances this aspect by introducing a suite of detailed Performance Metrics linked to specific interventions. Examples provided include tracking the number of patients engaged with the Health Coach program, beneficiaries of smoking cessation programs, average length of stay for certain procedures, and rates of antenatal care attendance. This move towards more granular performance measurement enables data-driven management, facilitates learning during implementation, and aligns with the principles of a “learning health system” that continuously improves based on evidence generated from practice [23].

In summary, the Saudi MoC complements its ambitious conceptual design with a detailed intervention list and a multi-faceted implementation strategy that considers phasing, context, enablers, and ongoing monitoring, drawing implicitly and explicitly on principles from implementation science.

## 4. Health Outcomes and Value Proposition

A central motivation for healthcare transformation initiatives globally, including the Saudi Model of Care (MoC), is the need to improve population health outcomes and ensure sustainable healthcare delivery. The MoC documentation explicitly addresses these imperatives and outlines a comprehensive value proposition focused on achieving better health for the population.

### 4.1. Health Outcomes Framework

The Saudi MoC has already demonstrated significant improvements in key health indicators, reflecting the effectiveness of the systems-based approach. According to the 2023 Statistical Yearbook, life expectancy at birth for Saudis has reached 78.8 years overall, with 78.1 years for males and 79.9 years for females [22]. This represents substantial progress toward the Vision 2030 target of an 80 year life expectancy. The Kingdom has achieved remarkable improvements in maternal and child health indicators. The maternal mortality ratio decreased from 14.0 deaths per 100,000 live births in 2010 to 9.42 deaths per 100,000 live births in 2022, representing a 33% decline. Similarly, the infant mortality rate decreased from 16.9 to 7.41 per 1000 live births between 2010 and 2022, showing a 56% improvement. The under-5 mortality rate also declined significantly from 19.5 to 10.05 per 1000 live births, representing a 48% reduction [22]. These improvements reflect the effectiveness of the integrated approach embedded in the Saudi MoC, particularly the Safe Birth system of care and the preventive care components. The systematic focus on maternal and child health, combined with improved access to quality healthcare services, has contributed to these notable achievements. The crude death rate has also shown improvement, declining from 2.81 per 1000 population in 2021 to 2.39 per 1000 population in 2023 [22]. This trend indicates overall improvements in population health and healthcare system effectiveness.

### 4.2. Comprehensive Value Proposition

The MoC documentation emphasizes that the model was designed to deliver comprehensive value across multiple dimensions. This value proposition aligns with established frameworks like the Quadruple Aim [33] and Porter’s value-based healthcare concepts [34]:

- Improved Health Outcomes: The MoC aims to improve the health of the population. Through targeted interventions, particularly those addressing leading causes of mortality like road traffic accidents and cardiovascular disease, the model projects significant health improvements, including increased average life expectancy in the Kingdom to 80 years by 2030, surpassing previous UN projections [16].
- Enhanced Patient Experience: Through its patient-centered design and specific interventions like the Health Coach Program, Virtual Self-Care Tools, and Enhanced Primary Care Services, the MoC aims to improve patient satisfaction, promote activation and engagement in self-care, and create a more navigable and supportive healthcare journey.
- System Efficiency and Integration: By addressing fragmentation and improving coordination, interventions such as National Referral Networks, Integrated Personal Health Records, and Case Coordination are intended to create a more seamless patient experience across different care settings, reduce duplication of services, and improve overall system efficiency.
- Workforce Development and Satisfaction: The model’s emphasis on team-based care, enhanced training, and potentially optimizing scopes of practice aims not only to build the necessary capacity but also to improve provider engagement and satisfaction, recognizing the critical role of the healthcare workforce in successful transformation.

### 4.3. Health Impact Framework

The MoC’s approach to improving health outcomes focuses on two primary mechanisms [16]:

- Prevention and Early Intervention: Decreasing the burden of disease through enhanced prevention, health promotion, and early detection. This includes reducing risk factors for chronic diseases, preventing accidents, and promoting healthy lifestyles.
- Care Quality and Integration: Improving health outcomes through better care coordination, evidence-based practices, and patient engagement. This encompasses streamlining pathways, reducing complications, and improving care continuity across settings.

The model particularly emphasizes improvements in areas such as maternal and child health, chronic disease management, and reduction in preventable mortality, reflecting national health priorities [16].

### 4.4. Implementation Considerations

While the MoC presents a comprehensive framework for improving health outcomes, several implementation considerations should be acknowledged:

- Time Horizon: Many health improvements, particularly from preventive interventions, may take years or decades to fully materialize. Long-term monitoring will be essential to capture these benefits.
- Attribution Challenges: In any complex, system-wide transformation involving multiple concurrent initiatives, definitively attributing observed changes in health outcomes to specific MoC interventions poses significant methodological challenges.
- Population Diversity: The impact may vary across different geographic regions, population subgroups, and socioeconomic contexts, requiring careful monitoring of equity in outcomes.
- Implementation Fidelity: Actual health outcomes will depend heavily on the quality and consistency of implementation across different settings and the successful development of enabling infrastructure.

Despite these considerations, the MoC provides a robust framework for pursuing improved population health. Future research should prioritize examining actual versus projected health outcomes, assessing the effectiveness of specific interventions, and evaluating the model’s long-term impact on population health as implementation progresses.

## 5. Discussion

This narrative review has examined Saudi Arabia’s National Model of Care (MoC) as a significant case study in systems-based healthcare transformation. Synthesizing information from key planning and revision documents [16,17], we have analyzed its conceptual underpinnings, the breadth of its interventions, its structured implementation strategy, and its projected health impact. The MoC emerges as a remarkably comprehensive and ambitious initiative designed to fundamentally reshape healthcare delivery in the Kingdom, aligning with the broader goals of Vision 2030.

### 5.1. Synthesis of Findings

The analysis confirms that the Saudi MoC is deeply rooted in systems thinking. Its multi-layered architectural framework explicitly integrates self-care, community resources, virtual platforms, and traditional clinical settings, moving beyond a purely facility-based view of healthcare. The unique organization around the patient-centric “Six Asks” provides a life-course perspective that structures care delivery around fundamental human needs rather than provider specialties. This conceptual design is operationalized through a detailed taxonomy of 42 cross-cutting and system-specific interventions, addressing areas from health promotion and prevention to acute care, chronic disease management, and end-of-life support. The implementation strategy further reflects systems principles through its phased rollout, emphasis on governance and capability building, planned contextual adaptations, reliance on key enablers like eHealth and workforce redesign, and incorporation of a monitoring framework with specific performance metrics.

### 5.2. Distinctive Features and Contributions of the Saudi MoC

The Saudi MoC offers several distinctive features that contribute valuable insights to the international discourse on healthcare transformation:

- Operationalizing Systems Thinking at National Scale: While many systems frameworks exist, the MoC provides a rare example of a nation attempting to translate these principles into a concrete, operational plan for its entire public healthcare system.
- Novel Organizing Principle (Six Asks): Structuring a national model around patient life-course needs rather than traditional administrative or specialty boundaries is an innovative approach that warrants further study regarding its impact on integration and patient experience.
- Explicit Integration of Non-Clinical Layers: The formal inclusion of activated individuals, healthy communities, and virtual care as core architectural layers represents a significant attempt to bridge the gap between clinical healthcare, public health, and self-management.
- Detailed Intervention Specification: The granular level of detail in defining the 42 interventions provides a practical blueprint that contrasts with more high-level strategic plans often seen in other contexts.
- Planned Contextual Adaptation: The foresight in planning for variations across geographic settings and special populations demonstrates a sophisticated understanding of implementation challenges in diverse environments.
- Alignment with Implementation Science: The conscious incorporation of elements like phased rollout, stakeholder engagement (in design), capability building, and performance monitoring reflects an appreciation for the science of translating policy into practice.

### 5.3. Critical Analysis: Strengths and Potential Challenges

The MoC exhibits considerable strengths in its design, notably its comprehensiveness, patient-centricity, emphasis on prevention and integration, and alignment with value-based healthcare principles. The detailed documentation and structured approach provide a solid foundation for transformation.

The MoC aligns with Berwick’s Triple Aim [24] by targeting outcomes, experience, and costs, and WHO health system building blocks [25] through workforce and governance focus; however, potential unintended consequences include digital divide exacerbating inequities in virtual care and workforce strain from rapid cluster consolidation.

However, the very ambition and complexity of the MoC also present significant potential challenges:

- Implementation Complexity: Coordinating 42 interventions across multiple layers, six systems of care, and ~20 regional clusters, while simultaneously developing five major enablers, represents an enormous implementation challenge requiring exceptional leadership, coordination, and sustained commitment.
- Dependence on Enablers: The success of many interventions is heavily contingent on progress in developing the enabling infrastructure, particularly eHealth systems (like the integrated PHR) and workforce redesign. Delays or shortcomings in these areas could significantly impede progress.
- Cultural and Behavioral Change: Shifting towards patient activation, prevention, and team-based care requires substantial changes in the attitudes and behaviors of both the public and healthcare professionals, which can be difficult and slow to achieve.
- Potential for Implementation Gaps: As with any large-scale plan, there is a risk of gaps between the intended design and actual implementation on the ground (implementation fidelity). Ensuring consistent adoption across diverse settings will be critical.
- Sustaining Momentum: The model requires long-term commitment and sustained investment in change management. Maintaining momentum across political and administrative transitions will be crucial.
- A notable implementation risk concerns the lag in payment reform and private-sector participation. Several MoC interventions presuppose the existence of value-based purchasing mechanisms and active private-sector engagement. However, while these are defined as enabling components in the MoC framework (2017 Overview, 2025 V2.0), they remain in early stages of development. At present, service delivery reforms are being rolled out, but financing reform and regulatory frameworks to incentivize value remain partly aspirational. This distinction between implemented elements and planned enablers must be recognized in assessing both progress and risks. To illustrate these linkages, Supplementary Table S2 presents a logic pathway connecting the Six Asks with exemplary interventions, required enablers, and intended outcomes.

### 5.4. Comparison with International Initiatives

Observed improvements in Saudi health indicators occurred during the same period as the Model of Care roll-out. Attribution to the Model of Care needs appropriate designs such as interrupted time series or quasi-experimental comparisons. Positioning the Saudi MoC within the global landscape of healthcare reform highlights its unique aspects. While sharing goals with initiatives like Accountable Care Organizations (ACOs) in the US (population health, value-based payment) [33], the MoC appears more prescriptive in its intervention design and national scope. It incorporates principles of integrated delivery seen in systems like Kaiser Permanente or Geisinger [34] but applies them across a national public system with a novel organizing framework (Six Asks). Compared to regional transformation efforts like the Canterbury Health System in New Zealand, known for its whole-system integration and focus on home-based care [18], the Saudi MoC operates at a larger, national scale. It also shares themes with Singapore’s Healthcare 2020 Masterplan, particularly the shift “beyond hospital to community” [35], but arguably presents a more encompassing life-course framework. The Saudi MoC seems distinctive in its combination of national scope, patient-centric life-course structure, detailed intervention taxonomy, and explicit integration of non-clinical layers.

### 5.5. Implementation Challenges and Success Factors

Drawing from implementation science and experiences elsewhere, several factors will likely be critical for the MoC’s success [18,19,35,36,37].

- Sustained Leadership and Vision: Consistent political and administrative leadership committed to the long-term vision is paramount.
- Effective Stakeholder Engagement: Ongoing engagement with patients, frontline clinicians, managers, and private sector partners is crucial for buy-in, co-design of solutions, and addressing emergent barriers.
- Adaptive Management and Learning: The ability of the system, particularly the cluster governance structures, to monitor progress, learn from experience, and adapt strategies flexibly will be vital.
- Alignment of Incentives: Ensuring that payment systems and non-financial incentives consistently support the desired changes in practice is essential.
- Balancing Fidelity and Adaptation: Effectively managing the tension between implementing the core components of the model faithfully while allowing necessary adaptations for local contexts requires careful oversight and evaluation.
- Capacity Building: Continuous investment in training and support for the workforce to develop the new skills and competencies required by the MoC.

### 5.6. Generalizability and Lessons Learned

While the Saudi MoC is tailored to the Kingdom’s specific context (Vision 2030, demographics, existing infrastructure), several principles and lessons may be generalizable:

- The value of a comprehensive, multi-layered conceptual framework that explicitly integrates clinical care with prevention, self-management, and community resources.
- The potential utility of structuring care around patient life-course needs as an alternative to traditional silos.
- The importance of detailed intervention planning alongside high-level strategy.
- The critical need to proactively plan for contextual adaptation within national programs.
- The necessity of co-developing key enablers (workforce, eHealth, payment) in parallel with service delivery changes.
- The essential role of a robust monitoring and evaluation framework informed by implementation science.
- Unique features include Vision 2030 financing and Hajj adaptations; generalizable elements encompass the Six Asks framework and phased rollout.

Policymakers in other countries undertaking large-scale transformation can learn from the MoC’s systematic design process and its explicit attention to implementation factors.

### 5.7. Policy Implications

The MoC has significant implications for health policy in Saudi Arabia and potentially beyond. It necessitates sustained policy focus on resource allocation towards primary care and prevention, acceleration of eHealth infrastructure development, implementation of value-based payment reforms, strategic workforce planning and training, and refinement of regulatory frameworks to support integrated care and new provider roles. The success of the MoC will likely depend heavily on the coherence and consistency of policy decisions across these domains over the long term.

### 5.8. Limitations

This review has several limitations. It draws primarily on Ministry of Health documents, which, while authoritative, may present the Model of Care in a favorable light and underemphasize challenges. Public access to performance indicators is limited, restricting opportunities for independent validation and raising the possibility of selective reporting. Temporal confounding is also likely, as improvements in health outcomes occurred during a period of multiple concurrent reforms, making attribution to the Model of Care alone difficult. Finally, patient and public perspectives were not incorporated, reducing the diversity of viewpoints represented. These constraints highlight the importance of future independent evaluations and mixed-method studies to assess the implementation and real-world impact of the Saudi Model of Care.

## 6. Conclusions

This narrative review has analyzed the Saudi Model of Care (MoC) as a significant example of systems-based healthcare transformation at a national scale. The MoC represents a comprehensive approach to addressing 21st century healthcare challenges through its multi-layered architecture, patient-centric “Six Asks” framework, and detailed taxonomy of 42 interventions. Its implementation strategy emphasizes governance, capability building, contextual adaptation, and continuous monitoring.

The Saudi MoC contributes valuably to healthcare transformation literature by demonstrating how systems thinking principles can be operationalized into a national blueprint. Its patient-centered design and implementation science approach offers insights for other nations pursuing large-scale reforms, particularly in shifting paradigms from hospital to home, treatment to prevention, and passive to activated participation.

The Kingdom recorded gains in life expectancy and maternal and child health during the reform period. These are national trends. Future work should test whether the Model of Care contributed to these trends using counterfactual designs and fidelity metrics. These achievements validate the potential of system-based approaches to improve population health and healthcare system performance. With a total population of over 32 million people distributed across diverse geographic regions, the Kingdom of Saudi Arabia has demonstrated that systems-based healthcare transformation is feasible even in large, complex healthcare systems. The model’s emphasis on integration, patient-centeredness, and value-based care aligns with international best practices while addressing the specific needs and contexts of the Saudi healthcare system.

Future research should employ interrupted time-series analyses of national indicators or realist evaluations to assess implementation mechanisms.

## Figures and Tables

**Table 1:** Taxonomy of interventions in the Saudi Model of Care (MoC), with official names and sources.

Category	Intervention Name (Official)	System of Care Association	Source (MoH Document)
Cross-Cutting (15)	Health in All Policies	All	2017 Overview; 2025 V2.0 Annex C
	School Education Programs	All	2017 Overview
	Virtual Education and Navigation Tools	All	2017 Overview
	Healthy Living Campaigns	All	2017 Overview
	Health Hotline Services	All	2017 Overview
	Enhanced Home Care Services	All	2025 V2.0
	Enhanced Primary Care Services	All	2025 V2.0
	National Referral Networks	All	2017 Overview
	Integrated Personal Health Records	All	2025 V2.0
	Systematic Data Collection	All	2025 V2.0
	Outcomes Monitoring	All	2025 V2.0
	Resource Optimization	All	2025 V2.0
	Health Research Programs	All	2017 Overview
	National Guidelines	All	2017 Overview
	Virtual Self-Care Tools	All	2025 V2.0
System-Specific (27)	Health Coach Program	Keep Well	2017 Overview
	Community-Based Wellness Programs	Keep Well	2017 Overview
	Workplace Wellness Programs	Keep Well	2017 Overview
	School Wellness Programs	Keep Well	2017 Overview
	Healthy Food Promotion	Keep Well	2017 Overview
	Health Edutainment Programs	Keep Well	2017 Overview
	Promoting the Saudi CDC	Keep Well	2025 V2.0
	One-Stop Clinics	Planned Procedure	2017 Overview
	Pathway Optimization	Planned Procedure	2025 V2.0
	Length of Stay Reduction Initiatives	Planned Procedure	2017 Overview
	Step-Down and Post-Discharge Services	Planned Procedure	2025 V2.0
	Resource Control Center	Urgent Problem	2017 Overview
	Urgent problem Clinics	Urgent Problem	2017 Overview
	Population-Based Critical Care Centers	Urgent Problem	2025 V2.0
	Premarital Screening	Safe Birth	2017 Overview
	Preconception Care Services	Safe Birth	2017 Overview
	Safe Birth Services	Safe Birth	2017 Overview
	National Birth Registry	Safe Birth	2025 V2.0
	Postnatal Care Services	Safe Birth	2017 Overview
	Well Baby Clinics	Safe Birth	2017 Overview
	Neonatal Care Services	Safe Birth	2025 V2.0
	Chronic Disease Screening	Chronic Condition	2017 Overview
	Case Coordination	Chronic Condition	2017 Overview
	Continuing Care Services	Chronic Condition	2025 V2.0
	Patient and Family Support	Last Phase of Life	2017 Overview
	Hospice Care Services	Last Phase of Life	2017 Overview
	Multidisciplinary Team Development	Last Phase of Life	2025 V2.0

Source: Adapted from Ministry of Health documents [16,17].

**Table 2:** Summary of Saudi Healthcare Clusters Heterogeneity (data from MoH Statistical Yearbook 2023 and GASTAT 2022).

Region/Clusters	Estimated Population (Millions)	Bed Density (per 1000)	Urban/Rural Coverage	Special Considerations
**Riyadh Province—3 clusters**	~8.2	2.8–3.5	Predominantly Urban	Capital region; high chronic disease burden
**Makkah Province—3 clusters**	~7.0	2.5–2.8	Urban/Rural Mix	Seasonal Hajj/Umrah surge
**Eastern Province—2 clusters**	~3.2	3.0–3.2	Predominantly Urban	Industrial and occupational health risks
**Al-Qassim Province—1 cluster**	~1.0	~2.2	Mixed	Agricultural region
**Madinah Province—1 cluster**	~2.3	~2.5	Urban/Rural Mix	Religious tourism
**Hail Province—1 cluster**	~0.74	~2.0	Mixed	Mountain/desert terrain
**Tabuk Province—1 cluster**	~0.88	~2.1	Mixed	Border security considerations
**Al-Jouf Province—1 cluster**	~0.60	~2.3	Predominantly Rural	Remote access challenges
**Northern Borders—1 cluster**	~0.37	~1.5	Predominantly Rural	Harsh desert climate
**Asir Province—1 cluster**	~2.1	~2.7	Urban/Rural Mix	Mountainous geography
**Najran Province—1 cluster**	~0.50	~1.8	Predominantly Rural	Border area
**Jazan Province—1 cluster**	~1.4	~2.4	Mixed	Coastal and agricultural

**Table 3:** Saudi MoC Implementation Strategy Mapped to CFIR Domains.

CFIR Domain	Saudi MoC Elements Related to the Domain	Baseline Values (Examples)
**Intervention Characteristics**	Detailed specification of 42 interventions providing clarity. Articulation of relative advantages (projected financial savings and health outcome improvements). Design includes adaptability for different contexts (geographic, special populations). Comprehensive documentation (e.g., 2017 MoC Overview, 2025 V2.0 Interventions) indicating design quality and packaging.	Baseline life expectancy: 74 years (2016), with a target of 80 years by 2030 [13,14,16]; Baseline NCD mortality: 73% of deaths (2016), with a target reduction through prevention to <10% probability of premature mortality by 2030 [7,15,16,23,24,25].
**Outer Setting**	Alignment with patient needs and expectations (structured around “Six Asks”). Responsiveness to external policies and mandates (explicit link to Saudi Vision 2030). Consideration of broader healthcare trends (implied response to global calls for transformation).	Baseline population: ~32.18 million (2022 census); projected growth to ~34.6 million by 2025 [26,27,28]; Baseline chronic disease prevalence high (e.g., diabetes ~18.5%), target improved management via Vision 2030 [29,30].
**Inner Setting**	Planned structural changes (e.g., Cluster governance, National Referral Networks). Focus on networks and communications (e.g., Integrated PHR, e-referral platforms). Aim to influence organizational culture (shift towards prevention, patient activation, team-based care). Consideration of implementation climate (planned resource allocation, future value-based incentives). Emphasis on readiness for implementation (leadership engagement, capability building programs).	Baseline hospital beds: Approximately 2.43 beds per 1000 population [31], target optimization via cluster reforms [16]; Baseline primary care utilization: Low (~20% of visits), target increase to gatekeeping role.
**Characteristics of Individuals**	Addressing knowledge and beliefs through training and development components. Building self-efficacy via capability building initiatives for staff. Acknowledging individual stage of change through a dedicated change management strategy.	workforce density: the physician workforce density in Saudi Arabia reached approximately 3.5 physicians per 1000 population, reflecting progress from earlier baseline levels, with Vision 2030 targeting further capacity-building and expanded professional roles [16,22].
**Implementation Process**	Formal planning process (outlined five-step approach). Engaging stakeholders (co-creation approach mentioned in initial design, ongoing communication emphasized). Structured execution strategy (phased rollout across clusters, prioritization of interventions). Mechanisms for reflecting and evaluating (Systematic Data Collection, Outcomes Monitoring, specific performance metrics in V2.0).	Primary health care (PHC) centers recorded over 100 million visits, with a steady increase compared to previous years (e.g., 15% rise from 2022), indicating improved access and capacity building [22]. Virtual consultations reached 50 million, including 20 million via the 937 hotline, 15 million through the Seha app (https://www.seha.sa/), and additional services via the X platform for prescriptions and consultations. Mobile clinic visits exceeded 5 million, supporting community-based care. Referrals totaled 2.5 million, facilitating integrated pathways. Home medical care services provided care to 1.2 million patients, reducing hospital burdens [22]. Virtual appointments numbered 30 million, enhancing timeliness. Average hospital length of stay was 4.5 days (down from 5.2 in 2022), and ICU stay averaged 3.8 days (down from 4.1), reflecting optimized resource use and efficiency gains with target reduction via phased metrics [16,17,18,19,20,21,22].

CFIR (Consolidated Framework for Implementation Research). The information is synthesized from MoC documentation [16,17], with statistical references and analyses presented in the review text.

## Data Availability

The original data presented in the study are openly available in https://www.health.sa/en/model-of-care (accessed on 1 August 2025).

## Supplementary Materials

The following supporting information can be downloaded at: https://www.mdpi.com/article/10.3390/healthcare13192453/s1, Supplementary Table S1. AACODS appraisal of key policy documents (2017 MoC Overview and 2025 MoC V2.0). Supplementary Table S2. Logic pathway linking the Six Asks, exemplar interventions, required enablers, and intended outcomes.
