# Supplementary material for: Exploring a Systems-Based Model of Care for Effective Healthcare Transformation: A Narrative Review in Implementation Science of Saudi Arabia’s Vision 2030 Experience
Source: Healthcare (Basel). 2025 Sep 27;13(19):2453. doi: 10.3390/healthcare13192453 (PMC12524123; doi:10.3390/healthcare13192453)
Supplement: Supplementary file 1 [file healthcare-13-02453-s001.zip › Supplementary Table S1.pdf]

Supplementary Table S1. AACODS appraisal of key policy documents

Criterion	2017 MoC Overview	2025 MoC V2.0
Authority	Saudi Ministry of Health, official publication	Saudi Ministry of Health, official publication
Accuracy	Peer-reviewed internal Ministry processes; aligned with Vision 2030 strategy	Revised based on stakeholder input; incorporates performance metrics
Coverage	National health system, six systems of care, 42 interventions	Expanded intervention detail, monitoring framework
Objectivity	Policy intent stated, potential positive framing acknowledged	Similar; transparent about planned vs. implemented elements
Date	2017	2025
Significance	Foundational national reform document	Current operational guide, key for ongoing transformation
