# Supplementary material for: Exploring a Systems-Based Model of Care for Effective Healthcare Transformation: A Narrative Review in Implementation Science of Saudi Arabia’s Vision 2030 Experience
Source: Healthcare (Basel). 2025 Sep 27;13(19):2453. doi: 10.3390/healthcare13192453 (PMC12524123; doi:10.3390/healthcare13192453)
Supplement: Supplementary file 1 [file healthcare-13-02453-s001.zip › Supplementary Table S2.pdf]

Supplementary Table S2. Logic pathway linking the Six Asks, exemplary interventions, required enablers, and intended outcomes.

Six Asks (Systems of Care)	Exemplar Interventions	Required Enablers	Intended Outcomes
Keep Well	Health Coach Program; School Wellness Programs	Workforce training; eHealth apps	Reduced NCD risk; healthier lifestyles
Urgent Problem	Urgent Care Clinics; Resource Control Center	Emergency protocols; referral networks	Faster access; reduced avoidable mortality
Planned Procedure	One-Stop Clinics; Length of Stay Reduction Initiatives	Case coordination; cluster governance	Shorter hospital stays; improved surgical outcomes
Safe Birth	National Birth Registry; Well Baby Clinics	Workforce (midwives, pediatricians); eHealth	Safer deliveries; lower maternal and child mortality
Chronic Condition	Case Coordination; Continuing Care Services	Integrated PHR; value-based payment	Improved chronic disease control; fewer complications
Last Phase of Life	Hospice Care Services; Multidisciplinary Teams	Workforce redesign; financing reform	Dignified end-of-life care; better patient-family support
